# Empathic Accuracy in Male Adolescents with Conduct Disorder and Higher versus Lower Levels of Callous-Unemotional Traits

**DOI:** 10.1007/s10802-016-0243-8

**Published:** 2016-12-29

**Authors:** N. Martin-Key, T. Brown, G. Fairchild

**Affiliations:** 1 Academic Unit of Psychology, University of Southampton, Southampton, UK; 2 Sussex Partnership National Health Service Foundation Trust, Worthing, UK; 3 Department of Psychology, University of Bath, Bath, UK

**Keywords:** Empathy, Affective empathy, Emotion recognition, Conduct disorder, Callous-unemotional traits

## Abstract

Adolescents with disruptive behavior disorders are reported to show deficits in empathy and emotion recognition. However, prior studies have mainly used questionnaires to measure empathy or experimental paradigms that are lacking in ecological validity. We used an empathic accuracy (EA) task to study EA, emotion recognition, and affective empathy in 77 male adolescents aged 13–18 years: 37 with Conduct Disorder (CD) and 40 typically-developing controls. The CD sample was divided into higher callous-emotional traits (CD/CU+) and lower callous-unemotional traits (CD/CU-) subgroups using a median split. Participants watched films of actors recalling happy, sad, surprised, angry, disgusted or fearful autobiographical experiences and provided continuous ratings of emotional intensity (assessing EA), as well as naming the emotion (recognition) and reporting the emotion they experienced themselves (affective empathy). The CD and typically-developing groups did not significantly differ in EA and there were also no differences between the CD/CU+ and CD/CU- subgroups. Participants with CD were significantly less accurate than controls in recognizing sadness, fear, and disgust, all *p*s < 0.050, *r*s ≥ 0.30, whilst the CD/CU- and CD/CU+ subgroups did not differ in emotion recognition. Participants with CD also showed affective empathy deficits for sadness, fear, and disgust relative to controls, all *p*s < 0.010, *r*s ≥ 0.33, whereas the CD/CU+ and CD/CU- subgroups did not differ in affective empathy. These results extend prior research by demonstrating affective empathy and emotion recognition deficits in adolescents with CD using a more ecologically-valid task, and challenge the view that affective empathy deficits are specific to CD/CU+.

**Electronic supplementary material:**

The online version of this article (doi:10.1007/s10802-016-0243-8) contains supplementary material, which is available to authorized users.

Empathy has been defined as the capacity to share the emotions displayed by others (Eisenberg and Miller 1987). For many years, researchers have sought to study the relationship between empathy and aggressive and antisocial behavior, working under the view that deficits in empathy may promote aggression, and particularly instrumental aggression (Blair 2005). It has been proposed that empathy is a multi-faceted phenomenon that can be fractionated into at least three forms: cognitive empathy (understanding others’ mental states/emotion recognition), affective empathy (feeling the same emotion as another person), and motor empathy (mirroring others’ body movements and facial expressions; Blair 2005). There is increasing evidence that individuals with Disruptive Behavior Disorders (DBDs) such as Conduct Disorder (CD) and Oppositional Defiant Disorder (ODD), show deficits in emotion recognition (Fairchild et al. 2009; Short et al. 2016) and affective empathy (de Wied et al. 2005; de Wied et al. 2012). Nevertheless, findings are inconsistent across studies and highly simplified stimuli or tasks have been used in many of these studies.

Studies employing questionnaire measures have consistently demonstrated lower levels of both cognitive and affective empathy in children and adolescents with DBDs relative to healthy controls (e.g., Anastassiou-Hadjicharalambous and Warden 2008; Cheng et al. 2012; Cohen and Strayer 1996; Jolliffe and Farrington 2004). However, contrary to models positing deficits in affective empathy in individuals with DBDs and high levels of callous-unemotional (CU) traits (an index of the affective and interpersonal aspects of psychopathy that can be assessed in children; Blair 2013), affective empathy is reported to be unrelated to levels of CU traits (e.g., Anastassiou-Hadjicharalambous and Warden 2008; Cheng et al. 2012).

Other commonly used measures of empathy include tasks assessing recognition of facial expressions of emotion (considered critical for cognitive empathy). Relative to healthy controls, children and adolescents with CD are reported to exhibit emotion recognition impairments, although it is currently unclear which emotions are affected. For example, when presenting morphed facial expressions, studies have found impairments in anger and disgust recognition in both males and females with CD, with additional impairments in happiness and fear recognition in males with CD (Fairchild et al. 2010; Fairchild et al. 2009). On the other hand, a study that investigated CD subjects’ ability to identify emotions from both faces and voices found deficits in happiness, fear, and sadness (but not anger) recognition in this group (Cadesky et al. 2000).

Studies investigating the effects of CU traits on facial emotion recognition have also yielded mixed findings, with some studies showing that CU traits are associated with deficits in recognizing facial expressions signalling distress (i.e., fear and sadness; Dadds et al. 2008; Fairchild et al. 2009, 2010), whilst other studies have reported *superior* fear recognition in those with high versus low levels of CU traits (e.g., Woodworth and Waschbusch 2008). Emotion recognition has also been measured using tasks involving the presentation of video clips (e.g., excerpts from films or documentaries). Here, findings have been even more mixed. Some studies have found no impairments in recognition of emotions in dynamic stimuli or video-clips in those with DBDs (e.g., de Wied et al. 2005; Schwenck et al. 2012), while one study found significant deficits in overall emotion recognition in adolescents with CD (Cohen and Strayer 1996), although data for individual emotions were not reported and it is therefore unclear whether some emotions were more affected than others.

Emotionally-laden video clips have also been employed to measure affective empathy responses, although there have been inconsistencies between studies in the operationalization of affective empathy. When affect matches (i.e., feeling the same emotion as another person) have been assessed, studies have found significantly fewer affect matches in children with DBDs relative to controls (e.g., de Wied et al. 2005). Other studies have focused solely on emotional intensity (e.g., Schwenck et al. 2012) or congruence (asking the participant whether he/she felt the same emotion or a similar valence as the target, irrespective of what the emotion was; e.g., Anastassiou-Hadjicharalambous and Warden 2008), finding that the DBD groups reported less intense emotions than the controls. It has also been shown that individuals high in CU traits exhibit greater impairments in affective empathy than those with low levels of CU traits, particularly for sadness (e.g., de Wied et al. 2012; Schwenck et al. 2012). Again, however, these findings have not been consistent, with some studies finding no effects of CU traits on affective empathy for sadness (e.g., Anastassiou-Hadjicharalambous and Warden 2008).

Taken together, it is evident that findings related to both cognitive empathy/emotion recognition and affective empathy in youths with DBDs have been inconsistent across studies. This is likely due to the wide range of definitions of empathy in the literature, as well as the different materials and tasks used in these studies. The kinds of static, grayscale stimuli depicting facial expressions used in most studies of facial emotion recognition do not resemble the facial stimuli we see in everyday life, whilst studies employing vignettes or films have often required participants to label an overall emotion and occasionally rate its strength and explain the reason for it. This dependence on requiring participants to make an overall judgement of the emotion, often through forced-choice procedures, means that it has not been possible to examine whether participants are able to continuously track changes in emotional intensity, which is a key skill in real-life social situations. Furthermore, selecting excerpts from television shows or scenarios portrayed by actors means that the emotion displayed in the clip is inevitably artificial and, further, that it is not possible to determine whether the targets were genuinely feeling the emotion they were portraying. Zaki et al. (2009) recently developed an Empathic Accuracy (EA) task that they believe overcomes many of these methodological issues. EA, defined as the capacity to correctly deduce the intensity and valence of the feelings being experienced by a target (Zaki et al. 2008; Zaki and Ochsner 2011), involves both mental state attribution (cognitive empathy/emotion recognition) and experience-sharing (affective empathy; Zaki and Ochsner 2011). Critically, the participant’s continuous ratings of emotional intensity during the clip are compared with the target’s own ratings of the emotions they experienced to yield an index of EA.

## The Current Study

In order to investigate whether participants are able to track changes in emotional intensity and address the issue of low ecological validity in previous work, as well as exploring recognition of dynamic stimuli and affective empathy, the present study employed a modified version of the EA task developed by Zaki et al. (2009). Rather than using undifferentiated positively- and negatively-valenced stimuli, we created video clips depicting each of the primary emotions, and we also asked participants to rate their own feelings after watching the video clips. Due to the difficulties of recruiting females with CD, as well as the fact that many of the previous studies in this area used male-only samples, the current study was restricted to male participants only. Our primary objective was to compare male adolescents with CD and typically-developing (TD) controls across these different measures of empathy. We also compared adolescents with CD and higher levels of CU traits (CD/CU+) with those with CD and lower levels of CU traits (CD/CU-) in terms of task performance. We predicted that participants with CD would be impaired in EA and would show emotion recognition and affective empathy deficits relative to TD controls. We also hypothesized that participants with CD/CU+ would show reduced EA, emotion recognition, and affective empathy relative to CD/CU- participants. We predicted that such deficits would be particularly marked for sadness and fear, given previous research showing disproportionate impairments in the processing of distress cues in those with high levels of CU traits (Dadds et al. 2006; Marsh and Blair 2008; Short et al. 2016).

## Method

### Participants

Thirty-seven male adolescents with CD and 40 TD male controls aged 13–18 years were recruited through Youth Offending Services and pupil referral units across Southampton and Hampshire via poster advertisements and referrals from case workers, and by sending out information packs to students at mainstream schools and colleges in the local area. Exclusion criteria included the following: Intelligence Quotient (IQ) < 70, as estimated using the two subtest version of the Wechsler Abbreviated Scale of Intelligence (WASI; Wechsler 1999), and the presence of Autism Spectrum Disorders (ASDs), psychosis, bipolar disorder or severe affective illness. All participants and the parents of those aged below 16 provided written informed consent to participate in the study, which was approved by the University Ethics Committee and the Southampton City Council and Hampshire County Council’s Children’s Services Research Governance Committees.

### Measures

#### The Schedule of Affective Disorders and Schizophrenia for School-Aged Children – Present and Lifetime Version

All participants were assessed for CD, ODD, attention-deficit/hyperactivity disorder (ADHD), major depressive disorder (MDD), generalized anxiety disorder (GAD), obsessive-compulsive disorder (OCD), post-traumatic stress disorder (PTSD), psychosis, and alcohol and substance use disorders using the Schedule of Affective Disorders and Schizophrenia for School-Aged Children - Present and Lifetime version (K-SADS-PL; Kaufman et al. 1997). The presence of ASDs was assessed using the ASD module of the unpublished DSM-5 version of the K-SADS-PL. Diagnostic interviews were carried out separately with participants and caregivers, and data were combined across informants such that a symptom was considered present if it was endorsed by either informant, as suggested by Kaufman et al. (1997). The inter-rater reliability of CD diagnoses was excellent (Cohen’s kappa =1.00).

#### The Inventory of Callous-Unemotional traits

CU traits were assessed using the self-report version of the Inventory of Callous-Unemotional traits (ICU; Frick 2003; Cronbach’s alpha in present sample = 0.78). Within the CD group, participants were categorised as CD/CU+ (*n* = 20) or CD/CU- (*n* = 17) using a median split procedure based on total ICU scores (median = 30; *M* = 30.05, *SD* = 8.81). Participants scoring ≥ 30 were classified as CD/CU+ while those scoring < 30 were classified as CD/CU-. This median value for the ICU, as well as the mean and SD values, are comparable to the mean scores and SD values reported in previous studies using the self-report version of the ICU (means ranging from 23.2 to 29.5, with SD values between 6.38 and 9.41; Feilhauer et al. 2012; Kimonis et al. 2008a: Kimonis et al. 2008b, Kimonis et al. 2016; Wolf and Centifanti 2014). Although this approach is common in the literature (Jones et al. 2010; de Wied et al. 2012; Schwenck et al. 2012), and there are no agreed cut-offs or norms on the ICU, there are limitations to using a median split procedure to dichotomise a continuous variable (i.e., reducing statistical power; MacCallum et al. 2002). In an attempt to address this issue, we also treated CU traits as a dimensional measure by testing for correlations between CU traits and EA, emotion recognition, and affective empathy within the CD group.

#### The Interpersonal Reactivity Index

To provide continuity with the previous literature on empathy in adolescents with DBDs, we also included a measure of dispositional empathy: the self-report Interpersonal Reactivity Index (IRI; Davis 1983; Cronbach’s alpha in present sample = 0.82).

#### Demographic Characteristics

Participants’ ethnicity was classified as either Caucasian or non-Caucasian, and their socioeconomic status (SES) was categorized as either high or low according to the parents’ occupations using the UK Office for National Statistics guidelines (ONS 2010).

#### Empathic Accuracy Task

This task was designed to assess whether participants could: a) track changes in the intensity of the target’s emotion (empathic accuracy; EA); and b) recognise the emotion displayed by the target after watching the full video clip (emotion recognition). We also investigated whether they reported experiencing the same emotion as the target (affective empathy). The task was adapted from a paradigm developed by Zaki et al. (2009). The creation of the stimulus materials, ratings of the stimuli by adults, and modifications to the task design are described in detail in the Online Supplementary Materials. In brief, actors (targets) were filmed talking about autobiographical experiences in which they had felt discrete primary emotions, rather than undifferentiated positive or negative emotions, and the continuous rating scale was used to rate changes in emotional intensity, rather than conflating intensity and emotional valence. The actors provided continuous ratings of the intensity of the emotions they experienced when filming the clips, while watching them directly afterwards.

### Procedure

**Fig. 1:**
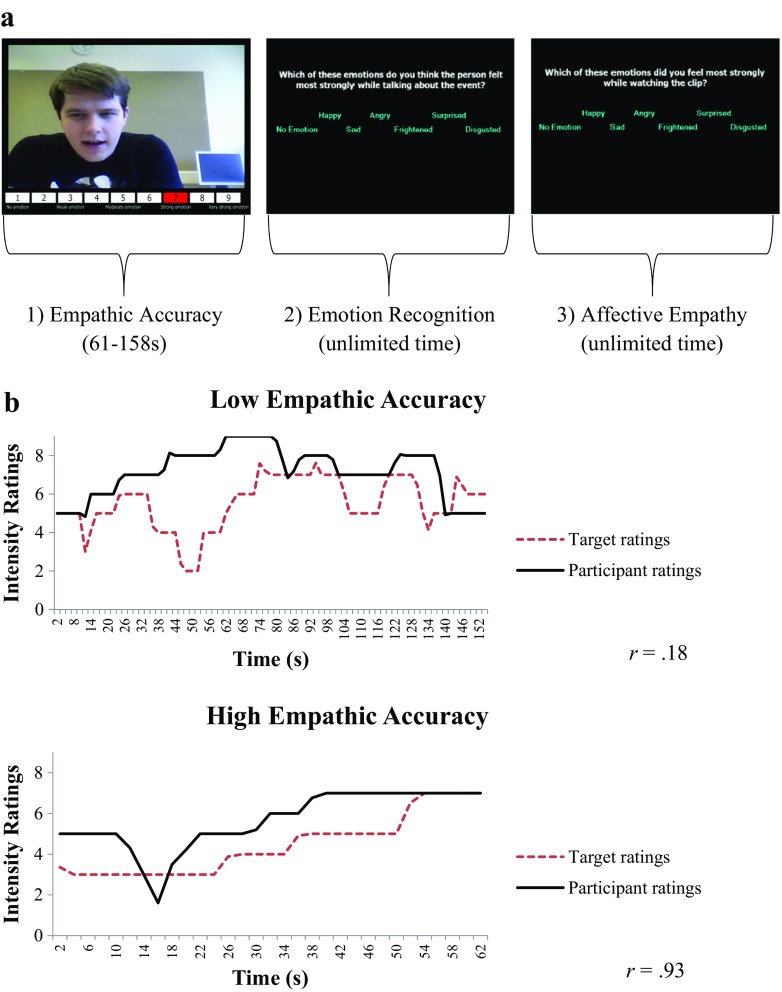
Schematic representation of a trial sequence of the empathic accuracy task (panel a) and examples of low and high correlations between the perceiver’s and the target’s continuous ratings of emotional intensity, i.e., low and high empathic accuracy (panel b)

Participants were asked to watch two practice clips to familiarize themselves with the task and rating scale, and then watched 12 test clips involving two instances of each of the following emotions: anger, happiness, sadness, disgust, fear, and surprise. These clips lasted between 61 and 158 s, with a mean length of 144 s. During the presentation of each video clip, participants were required to rate, on a continuous basis, the intensity of the emotions being experienced by the target on a nine-point rating scale (from 0 = *no emotion* to 9 = *very strong emotion*). We examined the correlations between the targets’ continuous ratings of the intensity of their emotions and the participants’ ratings of emotional intensity on the same scale (Fig. 1a). The correlation between the target’s and the participant’s continuous ratings formed the dependent measure of EA (see Fig. 1b for examples of low and high correlations). Following each clip, participants were asked to name the predominant emotion displayed in the video clip from a list of the six primary emotions. There was also an option of ‘*no emotion*’. Participants also named the emotion that they had experienced whilst watching the clip (again, with options of the six primary emotions and ‘*no emotion*’).

### Data Analytic Strategy

Continuous EA data were separated by clip. Mean ratings for each two-second period served as one data point (bin) in subsequent analyses. Participants’ ratings across all bins were correlated with the target’s own ratings. Correlations were then transformed using Fisher’s Z for all subsequent analyses, as recommended when averaging correlation coefficients (Silver and Dunlap 1987). Average correlations for each participant per emotion were then calculated. EA correlations were compared between groups using 2 (CD vs. control or CD/CU+ vs. CD/CU-) × 6 (sadness, happiness, fear, surprise, anger, disgust) mixed-design ANOVAs. For emotion recognition, participants’ performance accuracy was compared for each emotion separately using non-parametric statistical tests because the data were not normally distributed and could not be transformed to a normal distribution. Participants could receive scores of 0 (0/2 correct), 50 (1/2 correct) or 100% (2/2 correct) for each emotion. Emotion recognition scores for each emotion were compared between groups (CD vs. control and CD/CU+ vs. CD/CU-) using Mann-Whitney *U* tests, subject to the Holm-Bonferroni correction to correct for multiple comparisons (Holm 1979).

Similar procedures were used to compare the groups in terms of affective empathy as the data were not normally distributed; participants could receive scores of 0, 50, or 100% for affect matches for each emotion (i.e., same emotion as target in 0/2, 1/2, or 2/2 clips, respectively). Affective empathy scores for specific emotions were again compared between groups (CD vs. control; CD/CU+ vs. CD/CU-) using Mann-Whitney *U* tests, subject to the Holm-Bonferroni correction method. We also examined for effects of CU traits using a dimensional approach by testing for correlations between CU traits and EA, emotion recognition, and affective empathy (using either parametric or non-parametric bivariate correlations, as appropriate). Effect sizes are reported either as ‘r equivalent’ (Rosenthal and Rubin 2003) for the direct group comparisons (hereafter ‘*r*’; small ≥ 0.10, medium ≥ 0.30, large ≥ 0.50; Cohen 1988) or partial eta-squared (η_p_^2^) for the ANOVA analyses (small ≥ 0.01, medium ≥ 0.06, large ≥ 0.14; Cohen 1988). We also assessed the effects of potential confounds (i.e., group differences in IQ, SES, and psychiatric comorbidity). We first ran bivariate and point-biserial correlations between the variables that showed significant group effects and IQ, SES, and psychiatric comorbidity. Significant correlations were followed up by running multiple regression analyses to examine whether CD status or the potentially confounding variables were more important in explaining the observed group effects.

## Results

### Participant Characteristics

Demographic characteristics and rates of psychiatric comorbidity by group and between-group comparisons are presented in Table 1. The CD and control groups did not differ in age or ethnicity. However, the CD group had lower IQs than the control group, *t* (75) = −6.71, *p* < 0.001, *r* = 0.61, and members of the CD group were more likely to come from lower SES backgrounds than the control group, χ^2^ (1) = 13.89, *p* < 0.001, *r* = 0.85. Participants with CD had significantly higher levels of CU traits, *t* (75) = 3.47, *p* < 0.001, *r* = 0.40, and scored significantly lower than controls on all subscales of the empathy questionnaire (the IRI), except for personal distress. Approximately half (46%) of the CD participants had co-occurring ADHD diagnoses, and several CD participants had multiple comorbid disorders. However, 46% of the CD group had no current comorbid psychiatric disorders.

**Table 1:** Demographic characteristics and comorbidity: CD vs. TD group comparisons

	TD (*n* = 40)	CD (*n* = 37)	*p* value
	*M* (SD)	*M* (SD)	
Age (years)	16.20 (1.42)	16.03 (1.70)	0.639
Estimated IQ	104.18 (10.25)	89.27 (9.14)	<0.001
Callous-unemotional traits (ICU)	23.85 (6.84)	30.05 (8.81)	<0.001
Empathy questionnaire (IRI)			
Perspective-taking	15.55 (4.43)	12.19 (5.38)	0.008
Fantasy	14.38 (6.18)	10.95 (5.32)	0.009
Empathic concern	18.00 (3.97)	13.81 (4.94)	<0.001
Personal distress	11.28 (3.79)	11.27 (5.75)	0.543
	*n* (%)	*n* (%)	
Socioeconomic status ≠			
Higher	26 (65)	9 (24)	<0.001
Lower	8 (20)	21 (57)	
Missing	6 (15)	7 (19)	
Ethnicity			
Caucasian	34 (85)	33 (89)	0.598
Non-Caucasian	6 (15)	4 (11)	
Psychiatric comorbidity			
ADHD	0 (0)	17 (46)	-
Mood disorder	0 (0)	4 (11)	-
Anxiety disorder	0 (0)	5 (14)	-
Substance use disorder	0 (0)	5 (14)	-
Alcohol use disorder	0 (0)	2 (5)	-

≠ Estimated on the basis of parental occupation using the UK Office for National Statistics guidelines.

Key: *ADHD* attention-deficit/hyperactivity disorder, *CD* Conduct Disorder, *ICU* Inventory of Callous-Unemotional traits (self-report version), *IQ* intelligence quotient, *IRI* Interpersonal Reactivity Index, *SD* standard deviation, *TD* typically-developing

There was a significant difference between the CD/CU+ and CD/CU- groups in age, *t* (35) = 2.14, *p* = 0.041, *r* = 0.34, with the latter group being slightly younger than the former (see Table 2). There was also a difference in ethnicity, χ^2^ (1) = 5.28, *p* = 0.021, *r* = 0.70, with the CD/CU+ subgroup containing only Caucasian individuals, whereas the CD/CU- subgroup contained four non-Caucasian participants (out of 17). However, these subgroups were matched in IQ and SES. Confirming the effectiveness of the median split, the CD/CU+ group had higher levels of CU traits than the CD/CU- group, *t* (35) = 8.39, *p* < 0.001, *r* = 0.82. The CD/CU+ participants scored significantly lower on the perspective-taking subscale of the IRI than the CD/CU- participants, *t* (35) = −2.03, *p* = 0.047, *r* = 0.32, but there were no group differences for the other subscales. Rates of ADHD, mood and anxiety disorders were similar in the CD/CU- and CD/CU+ subgroups. Due to the relative absence of substance and alcohol use disorder comorbidity in the CD/CU- group, it was not possible to use statistical procedures to test for differences between subgroups in rates of these conditions.

**Table 2:** Demographic characteristics and comorbidity: CD/CU+ vs. CD/CU- group comparisons

	CD/CU-(*n* = 17)	CD/CU+(*n* = 20)	*p* value
	*M* (SD)	*M* (SD)	
Age (years)	15.41 (1.84)	16.56 (1.41)	0.041
Estimated IQ	87.88 (8.03)	90.45 (10.05)	0.402
Callous-unemotional traits (ICU)	22.35 (4.29)	36.60 (5.77)	<0.001
Empathy questionnaire (IRI)			
Perspective-taking	14.06 (4.93)	10.60 (5.35)	0.047
Fantasy	10.65 (4.68)	11.20 (5.92)	0.762
Empathic concern	14.82 (3.71)	12.95 (5.74)	0.361
Personal distress	11.29 (5.05)	11.25 (6.14)	0.262
	*n* (%)	*n* (%)	
Socioeconomic status ≠			
Higher	7 (41)	2 (10)	0.942
Lower	7 (41)	13 (65)	
Missing	3 (18)	5 (25)	
Ethnicity			
Caucasian	13 (76)	20 (100)	0.021
Non-Caucasian	4 (24)	0 (0)	
Psychiatric comorbidity*			
ADHD	6 (29)	11 (60)	0.192
Mood disorder	1 (24)	3 (30)	0.703
Anxiety disorder	1 (6)	4 (20)	0.358
Substance use disorder	0 (0)	5 (25)	-
Alcohol use disorder	0 (0)	2 (10)	-

≠ Estimated on the basis of parental occupation using the UK Office for National Statistics guidelines.

*Percentage values sum to more than 100% due to multiple comorbid disorders in some participants.

Key: *ADHD* attention-deficit/hyperactivity disorder, *CD/CU-* Conduct Disorder with lower levels of callous-unemotional traits, *CD/CU+* Conduct Disorder with higher levels of callous-unemotional traits, *ICU* Inventory of Callous Unemotional traits (self-report version), *IQ* intelligence quotient, *IRI* Interpersonal Reactivity Index, *SD *standard deviation

### Correlations between Dispositional Empathy (as Measured Using the IRI) and Empathic Accuracy, Emotion Recognition, and Affective Empathy

In order to establish the validity of the EA task, we tested for associations between the measures of interest (EA, emotion recognition, and affective empathy) and total IRI scores, as well as the perspective-taking, fantasy, empathic concern, and personal distress subscales. There were significant positive correlations between total IRI score and both overall EA, *r* = 0.23, *p* = 0.045, and overall affective empathy, *r* = 0.54, *p* < 0.001. We also found significant positive correlations between the perspective-taking, fantasy, and empathic concern subscales of the IRI and overall affective empathy, all *r*s > 0.35, *p*s < 0.010. No significant correlations were found between emotion recognition and total IRI score or the individual subscales, however.

### Empathic Accuracy: CD Vs. TD Group Comparisons

We assessed the participants’ ability to track changes in emotional intensity when viewing targets describing emotional autobiographical experiences, i.e., EA. There was no main effect of Group, *F* (1, 60) = 0.19, *p* = 0.661, η_p_^2^ = 0.010, or interaction between Group and Emotion, *F* (4.26, 255.49) = 1.20, *p* = 0.362, η_p_^2^ = 0.02, although the CD group achieved numerically lower scores for all emotions (see Table 3).

**Table 3:** Empathic accuracy descriptive statistics: CD vs. TD group comparisons

Emotion	TD (*n* = 40) Mean correlation (*r*) (SE)	CD (*n* = 37) Mean correlation (*r*) (SE)
Sadness	0.52 (0.03)	0.41 (0.03)
Happiness	0.51 (0.04)	0.50 (0.03)
Fear	0.51 (0.04)	0.44 (0.05)
Surprise	0.54 (0.05)	0.34 (0.06)
Anger	0.41 (0.04)	0.31 (0.05)
Disgust	0.49 (0.06)	0.37 (0.08)

Mean scores were transformed back to correlation coefficient scores (*r*) from Fisher’s Z for ease of interpretation.

Key: *CD* Conduct Disorder, *SE* standard error, *TD* typically-developing

### Empathic Accuracy: CD/CU+ Vs. CD/CU- Subgroup Comparisons

We also compared the CD/CU+ and CD/CU- subgroups in EA. Again, there was no main effect of Group, *F* (1, 25) = 1.47, *p* = 0.242, η_p_^2^ = 0.06, nor was there an interaction between Group and Emotion, *F* (5, 125) = 0.90, *p* = 0.491, η_p_^2^ = 0.04, although the CD/CU+ group achieved numerically lower scores than the CD/CU- group for all emotions (see Table 4). When treating CU traits as a dimensional measure, a significant correlation was found between CU traits and EA for sad clips, *r* = −0.35, *p* = 0.038, with elevated CU traits associated with a reduced ability to track changes in the intensity of sadness. However, none of the other correlations between CU traits and EA were significant.

**Table 4:** Empathic accuracy descriptive statistics: CD/CU- vs. CD/CU+ group comparisons

Emotion	CD/CU- (*n* = 17) Mean correlation (*r*) (SE)	CD/CU+ (*n* = 20) Mean correlation (*r*) (SE)
Sadness	0.47 (0.05)	0.37 (0.04)
Happiness	0.50 (0.05)	0.49 (0.04)
Fear	0.51 (0.09)	0.39 (0.05)
Surprise	0.39 (0.10)	0.30 (0.07)
Anger	0.33 (0.09)	0.30 (0.05)
Disgust	0.39 (0.14)	0.34 (0.07)

Mean scores were transformed back to correlation coefficient scores (*r*) from Fisher’s Z for ease of interpretation.

*﻿Key﻿: ﻿CD/CU-* Conduct Disorder with lower levels of callous-unemotional traits, *CD/CU+* Conduct Disorder with higher levels of callous-unemotional traits, *SE* standard error

### Emotion Recognition: CD Vs. TD Group Comparisons

**Fig. 2:**
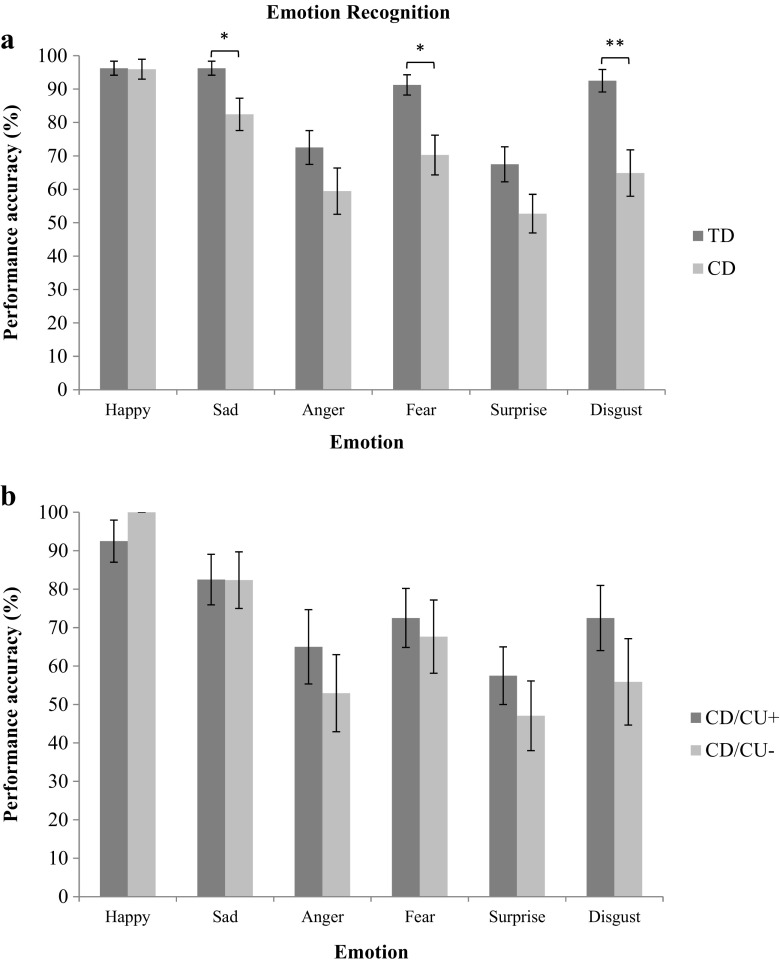
Emotion recognition scores for the typically-developing (TD) and Conduct Disorder (CD) groups (panel a), and the higher (CD/CU+) and lower (CD/CU-) callous-unemotional traits subgroups (panel b); error bars show +/−Standard Error. *Note:* The *p-*values are those obtained after applying the Holm-Bonferroni correction for multiple comparisons*;* *p < 0.050. **p < 0.010

We compared the TD and CD groups in terms of emotion recognition using Mann-Whitney *U* tests, subject to the Holm-Bonferroni correction for multiple comparisons. Participants in the CD group were significantly less accurate than controls in recognition of sadness, *U* = 572.50, *z* = −2.55, *p* = 0.044, *r* = 0.30, fear, *U* = 512, *z* = −2.86, *p* = 0.020, *r* = 0.30, and disgust, *U* = 478.50, *z* = −3.36, *p* = 0.006, *r* = 0.40 (see Fig. 2a). All of these group differences had medium effect sizes. Neither IQ, nor SES or psychiatric comorbidity was significantly associated with the recognition of these emotions, suggesting that these findings were not influenced by group differences in these variables.

### Emotion Recognition: CD/CU+ Vs. CD/CU- Subgroup Comparisons

We ran a similar analysis as described above for emotion recognition, but in this case comparing the CD/CU+ and CD/CU- subgroups. No significant group differences were found for any emotion (see Fig. 2b). When treating CU traits as a dimensional measure, no significant correlations were found between CU traits and emotion recognition performance.

### Affective Empathy: CD Vs. TD Group Comparisons

**Fig. 3:**
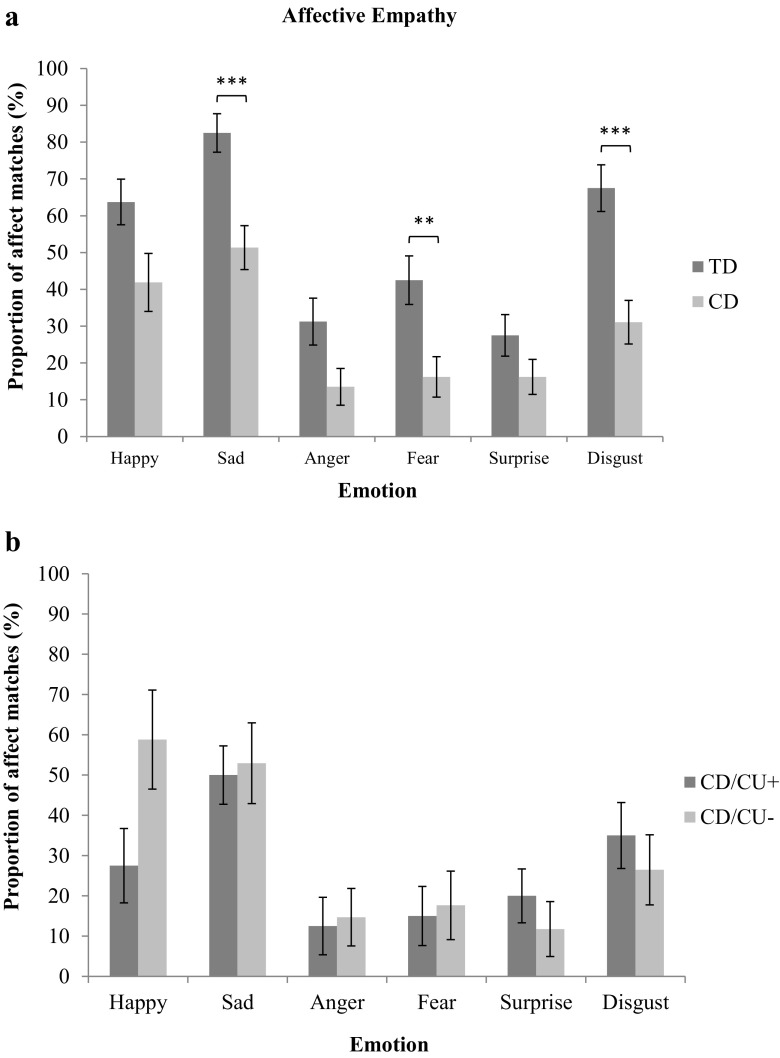
Affect matches to emotions displayed by targets in the typically-developing (TD) and Conduct Disorder (CD) groups (panel a) and the higher (CD/CU+) and lower (CD/CU-) callous-unemotional traits subgroups (panel b); error bars show +/−Standard Error. *Note:* The *p-*values are those obtained after applying the Holm-Bonferroni correction for multiple comparisons; **p < 0.010. ***p < 0.001

We analyzed the data for affective matches to the emotions displayed by targets. Mann-Whitney *U* tests were again used to test for group differences, applying the Holm-Bonferroni correction. Participants with CD reported significantly fewer affect matches than control participants when watching clips depicting sadness, *U* = 394, *z* = −3.89, *p <* 0.001, *r* = 0.41, fear, *U* = 476.5, *z* = −3.06, *p* = 0.010, *r* = 0.33, and disgust, *U* = 390, *z* = −3.79, *p* < 0.001, *r* = 0.40; see Fig. 3a. Again, all of these group differences had medium effect sizes.

When assessing for potential confounds, affective empathy for sadness, *r* = 0.40, *p* < 0.001, fear, *r* = 0.35, *p* = 0.002, and disgust, *r* = 0.36, *p* = 0.001, were positively correlated with IQ. Thus, separate multiple regression analyses were conducted for these emotions, with IQ and CD status as predictors of affective empathy. IQ was not a significant predictor of affective empathy for any of the emotions, all standardised *β*s < 0.23, *p*s > 0.100, indicating that the findings were not influenced by group differences in IQ. CD status was uniquely associated with reduced affective empathy for all of the emotions, all standardised *β*s > −0.24, *p* s < 0.050, with CD status accounting for ≥ 41.40% of the variance in affective empathy, all *R* ^*2*^s > 0.41, *F*s > 15.51, and *p*s < 0.050.

### Affective Empathy: CD/CU+ Vs. CD/CU- Subgroup Comparisons

We ran a similar non-parametric analysis as was described above to compare the CD/CU+ and CD/CU- subgroups in affective empathy. No differences between these subgroups were found for any emotion (see Fig. 3b). Likewise, when treating CU traits as a dimensional measure, no significant correlations were found between CU traits and affective empathy for any of the six emotions.

## Discussion

The aim of the present study was to assess empathic accuracy (EA), emotion recognition, and affective empathy in male adolescents with Conduct Disorder (CD) and higher versus lower levels of callous-unemotional (CU) traits, using a more ecologically-valid task than has been used previously. We note that issues with small sample sizes and limited statistical power must be taken into consideration when interpreting these findings. Relative to typically-developing (TD) adolescents, participants with CD showed deficits in emotion recognition and affective empathy when viewing intense and emotionally-evocative video clips depicting targets talking about real autobiographical experiences, and such difficulties were particularly marked for disgust, sadness, and fear. Contrary to our hypothesis, however, participants with CD were not significantly impaired in their ability to continuously track changes in emotional intensity (i.e., EA) relative to TD adolescents.

The present findings for emotion recognition of dynamic stimuli are broadly consistent with those obtained in studies using static images of facial expressions to investigate facial emotion recognition in adolescents with CD (Fairchild et al. 2009; Fairchild et al. 2010; Sully et al. 2015), and the current effect sizes were similar in size (i.e., medium) to those observed in previous studies. In particular, adolescents with CD have been reported to show deficits in fear and disgust recognition using tasks involving morphed facial expressions. The present study shows that such deficits are present even when the emotional stimuli contain visual, auditory, and linguistic information and the stimulus duration extends to multiple seconds or even minutes. Consequently, it seems likely that male adolescents with CD experience difficulties in recognizing or understanding others’ emotions in real-life social situations – therefore previous findings in this area were probably not explained by the use of highly artificial stimuli in the respective experimental paradigms.

The findings obtained for affective empathy were also very interesting. Adolescents with CD were found to show reduced affective empathy for sadness, fear, and disgust compared with TD controls. Again, all of these group differences had medium effect sizes. These results suggest that emotion recognition and affective empathy are related, consistent with a two-stage model in which cognitive empathy/emotion labelling precedes or provides a foundation for affective empathy (e.g., Batson 2009; Feshbach 1987), as impairments were seen for the same emotions as were identified in the emotion recognition analyses. In addition, these findings are in accordance with the notion that empathy is a multi-faceted phenomenon, which not only requires one to identify and understand others’ emotions/mental states, but also involves feeling the same emotion as the target. It seems intuitive that difficulties identifying emotions such as sadness, fear, and disgust might lead to deficits in affective empathy for these emotions. It could also be argued that the link between emotion recognition and affective empathy, where difficulties in the former affect the latter, influences the development of “moral socialization” (socialization via emotional learning). Indeed, Blair (1995) has proposed that TD children learn to desist from engaging in behaviors that harm others partly as a result of empathic processes (e.g., observing someone in pain or displaying fear evokes an empathic reaction, which is experienced as aversive and teaches the child not to perform the harmful/frightening action again). On the other hand, an individual who is less capable of identifying or sharing someone else’s feelings may not learn to desist from engaging in behaviors that cause harm to others (Blair 1995).

To address the second aim of the study, we directly compared the CD/CU+ and CD/CU- subgroups in terms of EA, emotion recognition, and affective empathy, to examine whether empathy deficits were more pronounced, or only present, in the CD/CU+ subgroup. Contrary to our hypotheses, there were no significant differences between these subgroups on any of the aforementioned measures. When treating CU traits as a dimensional measure, which is arguably a more powerful approach than using a median split, we found an inverse relationship between CU traits and EA for sadness, with higher levels of CU traits being associated with a reduced ability to track changes in the intensity of sadness. This is broadly consistent with previous studies showing impairments in the processing of distress cues in children and adolescents with CU traits (Dadds et al. 2006; Short et al. 2016), although it should be noted that there were no significant correlations between CU traits and any of the remaining 17 outcome measures.

Nevertheless, small sample sizes and accompanying issues with limited statistical power to detect differences between groups must be borne in mind when interpreting these null findings for the CD/CU+ vs. CD/CU- subgroup comparisons. Indeed, we acknowledge that the present findings may be considered surprising given previous work showing that empathy deficits are more pronounced in those with CD and elevated CU traits than those with lower levels of CU traits (Jones et al. 2010; Schwenck et al. 2012) and theories predicting that affective empathy deficits are uniquely related to CU traits (Blair 2005, 2013). On the other hand, these findings are consistent with previous research showing that the antisocial/lifestyle facet of psychopathy is more strongly related to deficits in empathy than the affective facet (Brook and Kosson 2013) and prior work with children with DBDs showing impaired empathy in both CU+ and CU- subgroups relative to TD children (de Wied et al. 2012). It is possible that previous studies have conflated the effects of CU traits and conduct problems, i.e., those with higher levels of CU traits have also tended to be higher in conduct problems, whereas in the present study we specifically examined the effects of CU traits *within* a sample of adolescents with diagnosable levels of conduct problems, i.e., CD.

Therefore, it is difficult to ascertain whether our null findings reflect the fact that CU traits have a limited impact on emotion recognition and affective empathy *within* CD populations, whether they are due to the fact that we had limited statistical power to detect differences between groups, or whether they are explained by the restricted range of CU traits in our sample (as few of our CD participants had very high levels of CU traits). Nevertheless, we note that the ICU score used to perform the median split in the present study is comparable to or higher than the mean scores reported in previous studies using the self-report version of the ICU, so the latter explanation seems unlikely.

### Strengths and Limitations

A major strength of this study was the use of a more ecologically-valid paradigm to simultaneously assess different forms of empathy and the use of video clips depicting discrete primary emotions, rather than just positive or negative emotions as in earlier studies using the EA task (e.g., Lee et al. 2011). The use of relatively naturalistic stimuli containing visual, auditory, and linguistic information means that our findings should be more applicable to real-life social situations than those obtained previously using artificial, highly-simplified stimuli. In addition, the fact that we obtained emotional intensity ratings *from the targets themselves* means that we were able to study EA for the first time in a CD population – this feature critically differentiates the present EA paradigm from other tasks of its type, such as the Multifaceted Empathy Test (Dziobek et al. 2008). In addition, the CD and control groups were well-characterized from a clinical perspective, psychiatric comorbidity was carefully assessed, and diagnostic information was obtained from multiple informants using standardized, semi-structured interviews.

However, this study also had a number of limitations. Firstly, given the relatively small sample size (*N* = 77), some of the present findings could reflect false positives. Alternatively, it could be claimed that the Holm-Bonferroni correction method may have been too conservative, thereby resulting in findings that represent false negatives. Either way, issues with statistical power should be borne in mind when interpreting these findings. Another important limitation relates to the EA task design. The movable scale used to provide emotion intensity ratings started at a default value of 5 (‘*moderate emotion’*). This may have discouraged participants from adjusting the scale upwards or downwards until pronounced changes in emotional intensity were detected. This design feature may have reduced the sensitivity of the task and restricted our ability to detect group differences in EA. Although this is the first study to obtain emotional intensity ratings from the targets themselves, the EA task relies on the *target’s initial ratings* of emotional intensity being accurate. For this reason, it may be advisable to use EA ratings collected from independent healthy samples as the reference point for calculating EA values in future studies.

Furthermore, it is important to note that some emotions were more difficult to empathise with than others. This was particularly true for clips depicting anger, fear, and surprise, where the majority of our participants did not report matching emotions. Although this could be a result of the clips used, it could be argued that some emotions (e.g., sadness) are more likely to evoke the same emotion in the perceiver, whereas other emotions, such as anger, might evoke alternative emotions in the perceiver, such as fear. Indeed, previous research has shown that the presentation of happy facial expressions induced happiness in the observer, whilst presenting angry facial expressions evoked fear (Dimberg 1988).

Another potential limitation of this study is the use of a median split procedure to define the CD/CU+ and CD/CU- subgroups. Although this approach is common in the literature (de Wied et al. 2012; Jones et al. 2010; Schwenck et al. 2012), and there are no agreed cut-offs or norms on the ICU, there are limitations to using a median split procedure to dichotomise a continuous variable, including losing or misrepresenting information about individual differences and reducing statistical power (MacCallum et al. 2002). Indeed, we only had adequate statistical power to detect medium and large effects, and were under-powered to detect small effects. In an attempt to address this issue, we also tested for correlations between CU traits and the different measures of empathy. Critically, these findings were largely consistent with those obtained using the median split approach.

It could be argued that using the self-report version of the ICU is problematic as it relies on the ability or motivation of young people to introspect and report on their own empathic capabilities. Although most studies revealing differences between CD/CU+ and CD/CU- individuals in empathic abilities have used parent- or teacher-report measures of CU traits, it is important to note that over 130 published studies have used self-report measures of CU traits or psychopathic traits in children and adolescents (see Frick et al. 2014). Many of these studies observed significant effects of self-reported CU traits. To our knowledge, there is no evidence to suggest that self-report measures of CU traits are less valid than parent-report or teacher-report measures, although we acknowledge that collecting data from multiple informants would have strengthened the study. Along similar lines, and given that no “gold standard” for assessing empathy currently exists, future studies might use multi-method approaches, such as assessing affective empathy via self-report, behavioral, and physiological outcome measures collected from the same individuals.

Finally, this study was restricted to male participants, so the present findings may not generalize to female samples. Consequently, future studies should investigate EA and other forms of empathy in females with CD using similar paradigms.

## Conclusion

This study extends previous research on empathy by demonstrating that, even when using rich and multi-sensory stimulus materials that are more ecologically-valid than those used in previous studies, male adolescents with CD still display significant impairments in emotion recognition and affective empathy – these deficits were particularly evident for sadness, fear, and disgust. To our knowledge, this is the first time that an EA task has been used with a population of this kind, and although we did not find any significant differences in EA between the CD and TD groups or between those with CD and higher versus lower levels of CU traits, further investigation of these issues with larger samples is merited. Experimental paradigms such as the EA task could potentially be used to assess empathy in clinical and forensic or judicial settings and to evaluate the effectiveness of interventions designed to enhance empathy in children and adolescents with disruptive behavior disorders.

## Electronic supplementary material

ESM 1 (DOCX 42 kb)
