# Supplementary material for: Empathic Accuracy in Male Adolescents with Conduct Disorder and Higher versus Lower Levels of Callous-Unemotional Traits
Source: J Abnorm Child Psychol. 2016 Dec 29;45(7):1385–97. doi: 10.1007/s10802-016-0243-8 (PMC5603649; doi:10.1007/s10802-016-0243-8)
Supplement: Supplementary file 1 — (DOCX 42 kb) [file 10802_2016_243_MOESM1_ESM.docx]

**Supplementary Materials for: *Empathic accuracy in male adolescents with Conduct Disorder and higher versus lower levels of callous-unemotional traits***

**The development and validation of stimuli for the empathic accuracy task**

**Method**

**Ethical approval**

Ethical approval for the filming of clips from targets and the pilot study with students was obtained from the University of Southampton’s Psychology Ethics Committee.

**Participants**

One group of participants served as actors (targets) by providing the stimulus materials to be used in the empathic accuracy task (i.e., video clips and continuous ratings of their own emotions). The majority of these actors were recruited from the Theatre Group at the University of Southampton, so they had some experience of acting on stage or in front of a video camera. We also recruited twelve Psychology students for the pilot study (the aim of this study was to validate the new version of the empathic accuracy task and the video stimuli depicting discrete primary emotions.

**Targets**

Targets were recruited through advertisements to male university students in the Academic Unit of Psychology and the Theatre Group at the University of Southampton. Participants were informed of the intended use of their video clips and were asked to sign two consent forms: one to consent to participate in the filming process and one to consent for the use of their video clips in the study. Six target participants contributed six video clips each, although clips from two of these targets were not used in the final experiment. The filming process took approximately two hours per participant, and the targets received £20 for taking part.

The mean age of targets was 23.5 years (SD = 4.76). Five targets described their ethnic origin as White British and one described himself as Asian. The clips that were ultimately used in the main study were all from White British targets.

**Pilot study**

Participants for the pilot study were recruited through an advertisement on a research participation website and posters displayed around the University of Southampton. Twelve participants with normal or corrected-to-normal vision and hearing were paid £10 or awarded course credits for a taking part in a 90 minute testing session.

The mean age of the pilot participants was 22.3 years (SD = 4.87). 9 of the pilot participants were female (75%). Ten (83%) were undergraduate students and two (17%) were postgraduate students.

**Procedure**

The procedures for collecting stimulus materials for the empathic accuracy (EA) task and designing the task itself were adapted from the pioneering work of Zaki, Bolger, and Ochsner (2008) and Zaki, Weber, Bolger and Ochsner (2009). The key methodological differences between our experiment and these previous studies are in the use of *discrete* primary emotions rather than just positive and negative emotions in the task stimuli, the use of a scale that covered a broader range of emotional intensities and did not confound valence and intensity, and the use of additional measures of cognitive and affective empathy as well as continuous intensity ratings to assess EA.

**Targets**

Individuals who responded to the advertisements met with the researcher and were informed of the aims of the research, their role and the intended audience for their video clips. They were asked to recall events where they had experienced one of the six basic emotions (happy, sad, frightened, surprised, angry, and disgusted) strongly and where it had been a relatively ‘pure’ emotion (i.e., it was not accompanied by other strong emotions). The participants wrote a brief description of the event, giving it a title of five words or less, and rated how strongly they had felt the emotion during the experience on a nine-point scale (from 1 = no emotion to 9 = very strong emotion, with 5 = moderate emotion). The participants then discussed the experience with the researcher, with emphasis on re-experiencing the emotion they had felt at the time. When participants reported feeling that they were re-experiencing the target emotion, they were filmed speaking about the event, although they were specifically asked to avoid naming the target emotion (e.g., ‘I felt sad.’). Immediately after filming, participants watched the clip and provided continuous ratings of the strength of their emotions on the nine-point scale. It was emphasised that they should rate how they had felt while speaking about the event during the filming process, rather than during the event itself. After viewing the clip, participants were asked to name the predominant emotion they had felt during filming and gave an overall intensity rating on the same nine-point scale. Before beginning the next clip, participants were given time for residual emotions to reduce in intensity. The procedure was employed for all six emotions; total filming time for each participant was approximately two hours. The order in which emotions were recalled and clips were filmed was pseudorandomised for each participant. Following their participation, targets were asked to provide written consent for their clips to be used in the final study and were thanked and debriefed.

**Pilot study participants**

The purpose of the validation study was to: (a) ensure that it was possible for student participants to identify the emotion being displayed by the target in the clips (our measure of cognitive empathy); (b) ensure that significant correlations between target and perceiver ratings could be achieved (empathic accuracy); and (c) determine whether perceivers tended to experience similar emotions to the ones displayed by the targets (affective empathy). These pilot data were used to select the clips for the final EA task that was used with adolescent participants.

Clips from five targets were included in the pilot study. Clips from one participant were excluded prior to piloting due to his low emotion intensity ratings when recalling experiences and during filming, and lack of variability in emotional intensity ratings at the post-recording stage.

Upon arrival at the lab, demographic information including the participant’s age, degree programme and year of study, home postcode and parental occupation were collected. Participants were introduced to the nine-point scale and observed the researcher using the scale to rate a training clip. It was emphasised that they should continuously rate the intensity of the emotion being experienced by the target while they were speaking, rather than during the event itself. Each clip was followed by three questions:

1. ‘Which of these emotions do you think the person felt most strongly while talking about the event?’ (options: happy, sad, frightened, angry, disgusted, surprised, no emotion)
2. ‘Which of these emotions did you feel most strongly while watching the clip?’ (options: happy, sad, frightened, angry, disgusted, surprised, no emotion)?
3. ‘How strongly did you feel that emotion?’ (rated using the same nine-point scale as used during the EA part of the task)

Participants viewed 31 clips in one of three pseudorandomised orders. No more than two clips from the same target or two clips depicting the same target emotion (e.g., sadness) were presented consecutively. Participants were offered regular breaks during the task to limit fatigue effects. They were debriefed at the end of the experiment regarding the aims and hypotheses of the study.

**Selection of stimulus materials**

The participants’ continuous ratings of emotional intensity of the clips on the nine-point scale (0 = no emotion, 5 = moderate emotion, 9 = very strong emotion) were correlated with the target’s own ratings of emotional intensity on the same scale to derive our measure of EA and identify clips yielding the highest EA values. Target and pilot participant data for each clip, separated by emotions, are presented in Supplementary Tables 1-6.

Data from targets and pilot study participants were collated and clips for the final EA task were selected based on the following criteria: (a) the target identified one of the primary emotions as the predominant emotion during the experience and this was rated at an intensity at or above the mid-point of the scale (i.e., 5 = moderate emotion); (b) the target reported re-experiencing the same primary emotion during filming and this was rated at an overall intensity at or above the mid-point of the scale (i.e., 5) during filming; (c) the majority of pilot participants accurately identified the target’s emotion; (d) the majority of pilot participants reported feeling an emotion in response to the film - where possible clips were selected that led to high levels of affective matches; and (e) the participants’ and targets’ continuous ratings were significantly correlated. Where more than two clips for an emotion from the same target met the above criteria, clips were selected which featured topics that were most likely to resonate with an adolescent audience. Two clips per emotion and two further training clips (one positive and one negative) were selected. The topics of the clips selected for the final study are presented in Supplementary Table 7.

Supplementary Table 1

*Target and pilot participant clip-rating data: Film clips portraying happiness*

| Target ID | Clip ID | Length (s) | During experience | |  | During filming | |  | Pilot ratings | | |
| --- | --- | --- | --- | --- | --- | --- | --- | --- | --- | --- | --- |
|  |  |  | Emotion | Intensity |  | Emotion | Intensity |  | Identified emotion (%) | Average correlation (r) | Felt emotion (%) |
| 1 | 6 | 45 | Happy | 8 |  | Happy | 8 |  | Happy (75), Surprised (25) | .749 (p<.001) | None (33.3), Happy (67.7) |
| 2* | 8 | 135 | Happy | 9 |  | Happy | 7 |  | Happy (100) | .442 (p<.001) | None (41.7), Happy (58.3) |
| 3 | 13 | 73 | Happy | 8 |  | Happy | 7 |  | Happy (100) | .165 (p=.330) | None (9.1), Happy (81.8),Sad (9.1) |
| 4 | 22 | 59 | Happy | 6 |  | Happy | 5 |  | None (9.1), Happy (63.6), Surprised (27.3) | .689 (p<.001) | None (27.3), Surprised (67.6), Happy (9.1) |
| 4* | 23 | 61 | Happy | 7 |  | Happy | 7 |  | Happy (100) | .951 (p<.001) | None (25), Happy (75) |
| 5 | 26 | 124 | Happy | 7 |  | Happy | 6 |  | Happy (100) | .620 (p<.001) | None (50), Happy (50) |

*Note:* * Clips selected for final study.

Supplementary Table 2

*Target and pilot participant clip-rating data: Film clips portraying sadness*

| Target ID | Clip ID | Length (s) | During experience | |  | During filming | |  | Pilot ratings | | |
| --- | --- | --- | --- | --- | --- | --- | --- | --- | --- | --- | --- |
|  |  |  | Emotion | Intensity |  | Emotion | Intensity |  | Identified emotion (%) | Average correlation | Felt emotion (%) |
| 1 | 2 | 52 | Sad | 7 |  | Sad | 7 |  | None (25), Sad (75) | .024 (p=.908) | None (33.3), Sad (66.7) |
| 2* | 7 | 152 | Sad | 7 |  | Sad | 5 |  | None (8.3), Sad (91.7) | .530 (p<.001) | None (8.3), Sad (91.7) |
| 3 | 14 | 61 | Sad | 9 |  | Sad | 6 |  | Sad (100) | -.099 (p=.598) | None (9.1), Sad (90.9) |
| 4 | 19 | 50 | Sad | 7 |  | Sad | 5 |  | None (16.7), Sad (83.3) | .858 (p<.001) | None (25), Sad (75) |
| 5* | 27 | 103 | Sad | 8 |  | Sad | 7 |  | Sad (100) | .673 (p<.001) | None (16.7), Sad (83.3) |

*Note:* * Clips selected for final study.

Supplementary Table 3

*Target and pilot participant clip-rating data: Film clips portraying anger*

| Target ID | Clip ID | Length (s) | During experience | | |  | | During filming | |  | Pilot ratings | | |
| --- | --- | --- | --- | --- | --- | --- | --- | --- | --- | --- | --- | --- | --- |
|  |  |  | Emotion | | Intensity | |  | Emotion | Intensity |  | Identified emotion (%) | Average correlation (r) | Felt emotion (%) |
| 1 | 4 | 60 | Angry | 9 | |  | | Angry | 8 |  | Angry (100) | .633 (p<.001) | None (25), Happy (8.3), Sad (33.3), Angry (16.7), Frightened (8.3), Surprised (8.3) |
| 2* | 11 | 158 | Angry | 8 | |  | | Angry | 7 |  | Angry (90.9), Disgusted (9.1) | .348 (p=.002) | None (9.1), Angry (45.5), Surprised (45.5) |
| 3 | 17 | 106 | Angry | 7 | |  | | Angry | 7 |  | Angry (54.5), Frightened (9.1), Disgusted (36.4) | .372 (p=.006) | None (18.2), Happy (9.1), Sad (9.1), Angry (18.2), Frightened (18.2), Surprised (9.1), Disgusted (18.2) |
| 4 | 24 | 63 | Angry | 8 | |  | | Angry | 6 |  | None (10), Angry (90) | .900 (p<.001) | None (20), Happy (10), Sad (30), Angry (20), Surprised (20) |
| 5* | 30 | 86 | Angry | 9 | |  | | Angry | 8 |  | None (16.7), Angry (66.7), Surprised (8.3), Disgusted (8.3) | .608 (p<.001) | None (25), Angry (33.3), Frightened (8.3), Surprised (16.7), Disgusted (16.7) |

*Note:* * Clips selected for final study.

Supplementary Table 4

*Target and pilot participant clip-rating data: Film clips portraying fear*

| Target ID | Clip ID | Length (s) | During experience | |  | During filming | |  | Pilot ratings | | |
| --- | --- | --- | --- | --- | --- | --- | --- | --- | --- | --- | --- |
|  |  |  | Emotion | Intensity |  | Emotion | Intensity |  | Identified emotion (%) | Average correlation (r) | Felt emotion (%) |
| 1 | 3 | 82 | Frightened | 8 |  | Frightened | 5 |  | None (9.1), Sad (36.4), Frightened (54.5) | .701 (p<.001) | None (27.3), Sad (27.3) Frightened (36.4), Surprised (9.1) |
| 2* | 9 | 129 | Frightened | 7 |  | Frightened | 6 |  | Sad (8.3), Frightened (91.7) | .762 (p<.001) | None (16.7), Sad (8.3), Frightened (66.7), Surprised (8.3) |
| 3 | 15 | 108 | Frightened | 6 |  | Frightened | 2 |  | None (16.7), Sad (8.3), Frightened (75) | .765 (p<.001) | None (25), Sad (50), Frightened (25) |
| 4 | 21 | 54 | Frightened | 7 |  | Frightened | 4 |  | None (16.7), Happy (8.3), Sad (8.3), Frightened (66.7) | .752 (p<.001) | None (58.3), Sad (8.3), Frightened (33.3) |
| 5* | 28 | 150 | Frightened | 8 |  | Frightened | 7 |  | None (18.2), Frightened (81.8) | .482 (p<.001) | None (27.3), Frightened (45.5), Angry (9.1), Surprised (18.2) |

*Note:* * Clips selected for final study.

Supplementary Table 5

*Target and pilot participant clip-rating data: Film clips portraying surprise*

| Target ID | Clip ID | Length (s) | During experience | |  | | During filming | |  | Pilot ratings | | |
| --- | --- | --- | --- | --- | --- | --- | --- | --- | --- | --- | --- | --- |
|  |  |  | Emotion | Intensity | |  | Emotion | Intensity |  | Identified emotion (%) | Average correlation (r) | Felt emotion (%) |
| 1 | 5 | 43 | Surprised | 99 |  | | Surprised | 7 |  | None (8.3), Happy (25), Surprised (66.7) | .760 (p<.001) | None (33.3), Happy (58.3), Angry (8.3) |
| 2* | 10 | 133 | Surprised | 7 |  | | Surprised | 5 |  | None (9.1), Sad (9.1), Surprised (81.8) | .849 (p<.001) | None (27.3), Sad (45.5), Surprised (27.3) |
| 3* | 16 | 77 | Surprised | 8 |  | | Surprised | 2 |  | None (16.7), Sad (8.3), Happy (8.3), Surprised (66.7) | .465 (p<.001) | None (33.3), Sad (41.7), Surprised (25) |
| 4 | 20 | 97 | Surprised | 7 |  | | Surprised | 6 |  | Happy (91.7), Surprised (8.3) | .747 (p<.001) | None (16.7), Happy (66.7), Surprised (16.7) |
| 5 | 29 | 104 | Surprised | 6 |  | | Surprised | 6 |  | Sad (50), Angry (16.7), Surprised (33.3) | .486 (p<.001) | None (16.7), Sad (50), Angry (16.7), Surprised (16.7) |

*Note:* * Clips selected for final study. 99 = missing data.

Supplementary Table 6

*Target and pilot participant clip-rating data: Film clips portraying disgust*

| Target ID | Clip ID | Length (s) | During experience | |  | During filming | |  | Pilot ratings | | |
| --- | --- | --- | --- | --- | --- | --- | --- | --- | --- | --- | --- |
|  |  |  | Emotion | Intensity |  | Emotion | Intensity |  | Identified emotion (%) | Average correlation | Felt emotion (%) |
| 1 | 1 | 42 | Disgusted | 8 |  | Disgusted | 7 |  | Surprised (9.1), Disgusted (90.9) | .147 (p=.513) | None (9.1), Frightened (9.1), Disgusted (81.8) |
| 2* | 12 | 99 | Disgusted | 6 |  | Disgusted | 6 |  | Disgusted (100) | .879 (p<.001) | Disgusted (100) |
| 3 | 18 | 98 | Disgusted | 6 |  | Disgusted | 6 |  | None (9.1), Happy (9.1), Disgusted (81.8) | .770 (p<.001) | None (18.2), Disgusted (81.8) |
| 4 | 25 | 47 | Disgusted | 7 |  | Disgusted | 7 |  | Happy (9.1), Frightened (9.1), Disgusted (81.8) | .648 (p=.001) | None (27.3), Surprised (9.1), Disgusted (63.6) |
| 5* | 31 | 90 | Disgusted | 7 |  | Disgusted | 7 |  | Disgusted (100) | .881 (p<.001) | None (9.1), Happy (18.2), Disgusted (72.7) |

*Note:* * Clips selected for final study.

Supplementary Table 7

*Topics of film clips selected for the main study on the basis of the pilot results*

| Emotion (clip number) | Content |
| --- | --- |
| Training - Angry (4) | Being bullied |
| Training - Happy (23) | Passing an important exam |
| Happy (8) | A football team winning an important game |
| Happy (22) | Going on a favourite holiday |
| Sad (7) | Death of a grandparent |
| Sad (27) | Family break-up and depression |
| Angry (11) | Being accused of a crime you didn’t commit |
| Angry (30) | Being attacked outside a nightclub |
| Frightened (9) | Returning home to discover a break-in |
| Frightened (28) | Being followed by a group of males in a train station |
| Surprised (10) | Finding out about a friend’s bad luck |
| Surprised (16) | A romantic relationship ending unexpectedly |
| Disgusted (12) | Finding rotting food in the bin |
| Disgusted (31) | Eating unfamiliar, horrible tasting food |

**References**

Zaki, J., Bolger, N., & Ochsner, K. (2008). It takes two: The interpersonal nature of empathic accuracy. *Psychological Science, 19*(4), 399-404. doi: 10.1111/j.1467-9280.2008.02099.x

Zaki, J., Weber, J., Bolger, N., & Ochsner, K. (2009). The neural bases of empathic accuracy. *Proceedings of the National Academy of Sciences of the United States of America, 106*(27), 11382-11387. doi: 10.1073/pnas.0902666106
